# Dietary Intake, Body Composition, and Menstrual Cycle Changes during Competition Preparation and Recovery in a Drug-Free Figure Competitor: A Case Study

**DOI:** 10.3390/nu8110740

**Published:** 2016-11-20

**Authors:** Tanya M. Halliday, Jeremy P. Loenneke, Brenda M. Davy

**Affiliations:** 1 Department of Human Nutrition, Foods, and Exercise, Virginia Tech, Blacksburg, VA 24060, USA; bdavy@vt.edu; 2 Division of Endocrinology, Metabolism and Diabetes, School of Medicine, University of Colorado Anschutz Medical Campus, Aurora, CO 80045, USA; 3 Kevser Ermin Applied Physiology Laboratory, Department of Health, Exercise Science and Recreation Management, University of Mississippi, University, MS 38677, USA; jploenne@olemiss.edu

**Keywords:** bodybuilders, physique athletes, competition preparation, competition recovery, dieting, energy availability, amenorrhea

## Abstract

Physique competitions are events in which competitors are judged on muscular appearance and symmetry. The purpose of this retrospective case study was to describe changes in dietary intake, body mass/composition, and the menstrual cycle during the 20-week competition preparation (PREP) and 20-week post competition recovery (REC) periods of a drug-free amateur female figure competitor (age = 26–27, BMI = 19.5 kg/m^2^). Dietary intake (via weighed food records) and body mass were assessed daily and averaged weekly. Body composition was estimated via Dual-energy X-ray absorptiometry (DXA) and 7-site skinfold measurements. Energy intake, body mass and composition, and energy availability decreased during the 20-week PREP period (changes of ~298 kcals, 5.1 kg, 6.5% body fat, and 5.4 kcal/kg fat free mass, respectively) and returned to baseline values by end of the 20-week REC period. Menstrual cycle irregularity was reported within the first month of PREP and the last menstruation was reported at week 11 of PREP. Given the potentially adverse health outcomes associated with caloric restriction, future, prospective cohort studies on the physiological response to PREP and REC are warranted in drug-free, female physique competitors.

## 1. Introduction

Physique competitions (bodybuilding, figure, and bikini) are unique athletic events in which competitors are judged on muscular appearance and symmetry rather than physical performance. In preparation for these contests competitors aim to decrease fat mass while maintaining lean mass through a combination of prolonged (≥12 weeks) caloric restriction, resistance training, and aerobic exercise [1,2]. Currently, no evidence-based dietary guidelines exist for physique athletes to achieve body mass/composition goals for competition, or to re-gain appropriate levels of fat mass following competition, particularly in a manner that preserves (or at least minimizes risks to) overall health [1,3]. This may contribute to the large number of preparation strategies implemented by coaches and athletes, some of which may be dangerous (extremely low caloric intakes, reliance on un-tested supplements, extreme dehydration, etc.) [4,5,6,7,8,9]. Healthcare professionals working with these understudied athletes will need to understand the culture and associated constraints of the sport in order to assist competitors in developing nutrition strategies to support their training and competition goals.

Previous research on physique athletes is limited and has mainly focused on male competitors, female competitors using anabolic steroids, and/or the competition preparation (PREP) phase only [1,3,10,11,12,13,14,15,16,17]. Furthermore, the published literature on female physique competitors is limited by: (1) low methodological quality; (2) inadequate description of competition phase; and (3) being dated (e.g., published in the 1980s and 1990s when top-level competitors had lower body masses, and fewer competition categories existed [1,3]. Thus the data may be less applicable to current day physique competitors). Recent case studies of male physique competitors [11,18,19,20] have provided empirical evidence on the nutritional and exercise regimens, and the associated metabolic and physiological responses of these athletes. To our knowledge, no studies have provided a detailed account of both the PREP and competition recovery (REC) phases in drug-free female competitors. Given the potential health implications (e.g., female athlete triad) of obtaining a low level of fat mass through caloric restriction and exercise [21,22], evaluation of these athletes is warranted. To address gaps in the literature, the purpose of this case study was to describe changes in dietary intake, body mass and composition, and the menstrual cycle in a drug-free, female, figure competitor during both the PREP and REC periods.

## 2. Materials and Methods

This case study was considered exempt from Institutional Review Board review and approval. It was conducted and prepared in accordance with the Health Insurance Portability and Accountability Act.

### 2.1. Subject and Timeline Overview

**Figure 1:**
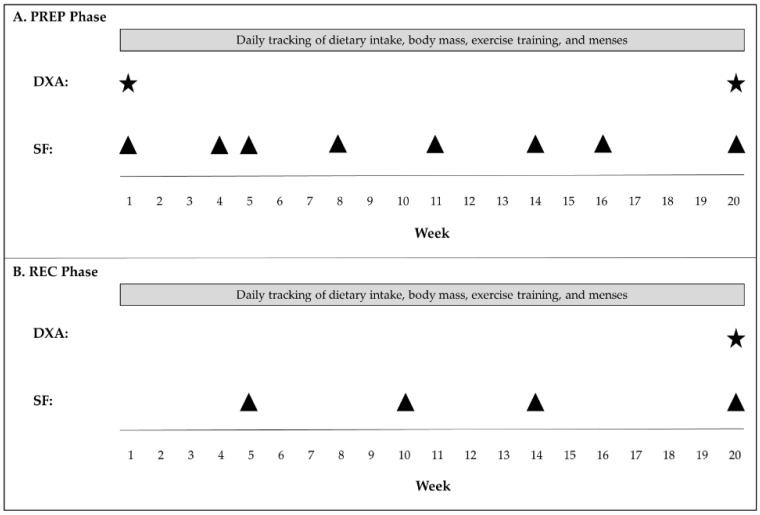
Timeline of Study Measurements (**A**) PREP and (**B**) REC phases. PREP: Competition preparation; REC: Competition recovery; DXA: Dual-energy X-ray absorptiometry; SF: Skinfolds.

The subject (26–27 years; BMI: 19.4 kg/m^2^; body fat: 15%) was a Caucasian, drug-free, amateur figure competitor preparing for her first competition. The subject did not take any medications, including oral contraceptives, during the PREP or REC periods. The 20-week competition PREP and 20-week REC timeline for this competitor, including nutrition and exercise training programs were developed in collaboration with a contest preparation coach who is a certified personal trainer and professional male natural bodybuilder with 20 years of competition and coaching experience. Alterations to the program were determined based on body composition changes and subjective assessment of physique during posing practices. An overview of the timeline for study measurements is presented in Figure 1. In addition, the subject returned for assessment of body mass and composition (DXA) 32 weeks after the competition (i.e., 1 year since the initiation of PREP) and when menses resumed, 71 weeks post competition.

### 2.2. Dietary Intake

The subject electronically tracked dietary intake via weighed food records, using a commercially available digital food scale (Soehnle Optica^®^) to the nearest gram throughout PREP. Following the competition, the subject was less motivated to maintain a rigid diet and track intake as diligently. Thus, the 20-week REC period contains estimates of weekly macronutrient and caloric intake from a combination of weighed records and food diary estimates. Nutrient information was obtained from the USDA National Nutrient Database [23] or product-specific nutrition facts panels. Daily nutrient intake information (total kcals, macronutrient (g and %), and fiber (g)) was averaged each week.

### 2.3. Body Mass and Composition

The subject electronically tracked body mass daily on a commercially-available home scale (Health O Meter Professional^®^) throughout PREP and REC. Daily body masses were averaged each week. Body composition was assessed just before the PREP period began, the week of the competition (week 20 PREP), and at week 20 of the REC period via dual-energy X-ray absorptiometry (DXA; pre-PREP using Lunar Prodigy Advance, GE Medical Systems, software version 8.10e, Madison, WI, USA; and remainder of scans using Lunar iDXA, GE Medical Systems, software version enCORE 15, Madison, WI, USA model due to equipment upgrading in our laboratory) performed by a trained research technician licensed as a Radiologic Technologist-Limited in the state of Virginia. Skinfold thickness was measured 8 times during the 20-week PREP period and 4 times during the 20-week REC period via 7-site skinfold measurements according to ACSM guidelines [24] and using Jackson-Pollock generalized skinfold equation for body density [25] and the Siri equation for estimating body fat [24] by the subject’s contest preparation coach.

### 2.4. Exercise Training

The subject recorded (paper/pen) exercise training daily for the duration of PREP and REC periods. Exercise Energy Expenditure (EEE) was estimated using the 2011 Compendium on Physical Activity [26].

### 2.5. Energy Availability

Before and at weeks, 1, 10, and 20 of PREP and weeks 10 and 20 of REC energy availability (EA) ((energy intake (kcals)-EEE (kcals))/fat-free mass (FFM) (kg)) [27] was calculated from the dietary intake record, exercise training record, and estimated FFM of the corresponding week. The established threshold of 30 kcal/kg was used as the reference level for comparing this subject’s EA to the level below which adverse health outcomes have been detected [21].

### 2.6. Menstrual Cycle

Menses was tracked (paper/pen calendar) and reported by the subject for PREP and REC phases. 

## 3. Results

### 3.1. Dietary Intake

The subject’s diet during PREP and REC consisted of 2 days of high carbohydrate intake (~180–230 g/day), 3 days of moderate carbohydrate intake (~150–180 g/day), and 2 days of low carbohydrate intake (~100–150 g/day) each week. High carbohydrate intake days occurred on lower body resistance training days and low carbohydrate intake days occurred on off or low-intensity cardio training days. A sample daily weighed food record is presented in Table 1, representing typical food choices and portions consumed during both PREP and REC. Dietary supplement intake included: whey and casein protein powders, which were calculated into daily caloric and protein intake totals; and 5 g/day of creatine monohydrate from weeks 11 to 20 of PREP.

**Figure 2:**
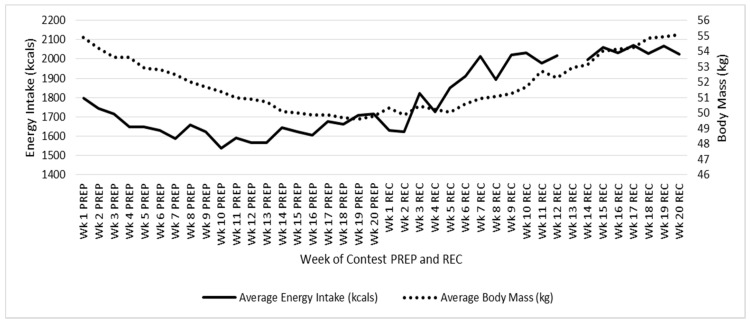
Changes in Energy Intake and Body Mass during Competition Preparation and Recovery. PREP: Competition preparation; REC: Competition recovery.

Changes in averaged weekly caloric intake are presented in Figure 2 for PREP and REC. Habitual energy and macronutrient intake at baseline (i.e., before PREP), weeks 1, 10, and 20 of PREP, and weeks 10 and 20 of REC are listed in Table 2.

### 3.2. Body Mass and Composition

Changes in average weekly body mass are presented in Figure 2 for PREP and REC. Body mass decreased from 54.9 kg at Week 1 of PREP to 49.8 kg by Week 20 of PREP, and then increased to 55.1 kg by Week 20 REC. Body fat (assessed via DXA) decreased from 15.1% (8.3 kg) at baseline to 8.6% (4.3 kg) by Week 20 of PREP. Lean mass was maintained at 44.3 kg pre and post PREP (80.7% and 89% lean mass, respectively). By Week 20 REC, percent body fat had returned to baseline at 14.8%. By Week 32 of REC (e.g., 1 year since initiation of PREP), body mass had increased to 57.3 kg and body fat to 20%. By Week 71 REC (when menses resumed) body mass was 56.1 kg and body fat had been maintained at 20%.

**Figure 3:**
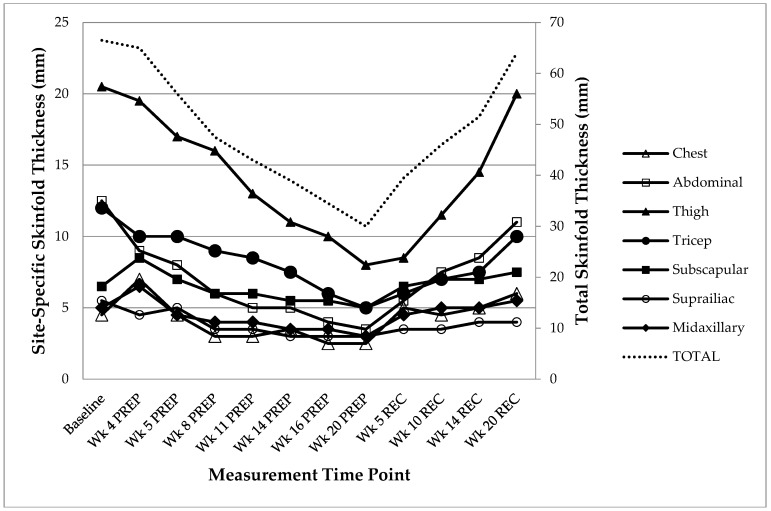
Total and Site-Specific Skinfold Thickness Changes. Wk: Week; PREP: Competition Preparation; REC: Competition recovery.

Total and site-specific skinfold thickness changes during PREP and REC are presented in Figure 3. Total skinfold thickness decreased from 66.5 mm at Baseline to 30 mm by the week of competition (Week 20 PREP) (corresponding to a decrease from 14.8% to 8.3% body fat, indicating concordance with the DXA results). Skinfold thickness steadily increased in the REC period and returned to baseline (64 mm) by Week 20 REC.

### 3.3. Exercise Training

Exercise training during PREP consisted of a high-volume resistance training program 4–5 days/week (training all major muscle groups of the upper and lower body 2–3 days/week), brief (e.g., 10–30 min) high-intensity interval training 1–2 day(s)/week, and longer (e.g., 45–120 min) aerobic exercise session 1 day/week. This training regimen resulted in an EEE of 484, 459, and 440 kcal/day at weeks 1, 10, and 20 of PREP, respectively. Exercise training during REC consisted of a high-volume resistance training program 3–4 days/week, brief (10–30 min) high-intensity interval training 1–2 day(s)/week, and a longer (45–60 min) aerobic exercise session 1 day/week. This training regimen resulted in an EEE of 355 and 378 kcal/day at weeks 10 and 20 of REC, respectively.

### 3.4. Energy Availability and Menstrual Cycle

Prior to PREP and at weeks 1, 10, and 20 of PREP, EA was 32.7, 28.2, 23.2, and 27.3 kcal/kg FFM, respectively. At weeks 10 and 20 of REC, EA was estimated to have increased to 36.5 kcal/kg and 35.1 kcal/kg FFM, respectively. Our subject reported a habitual cycle length of ~42 days without use of hormonal birth control for several years prior to engaging in competition preparation. Menstrual cycle irregularity (spotting between typical menses) was reported within the first month of PREP and the last menstruation was reported at week 11 of PREP. Menses did not resume until 71 weeks following the competition.

## 4. Discussion

This case study provides a detailed and comprehensive examination of the dietary and exercise habits, and the associated alterations in body mass, body composition, EA, and menses during both the PREP and REC phases for a drug free, female figure competitor. The major finding from this investigation was that caloric restriction, low EA, and decreased fat mass led to loss of menses early in the PREP phase. Despite a return to baseline levels of energy intake, EA, and fat mass during the REC phase, resumption of menses was delayed. In addition, this investigation provides insight on the diet-related culture of the sport which is vital for healthcare professionals working with these clients to be familiar with.

### 4.1. Dietary Intake

#### 4.1.1. Energy Intake

Competition PREP for our subject consisted of a gradual decrease in energy intake for the initial 10-weeks and then a gradual increase back to week 1 PREP energy intake by week 20 PREP (i.e., week of competition). Our subject’s habitual (2010 kcals/day) and PREP energy intake (low of 1541 kcals/day at week 10) were greater than those previously reported in female physique competitors (average of 1636 and 1214 kcals/day, respectively) [1]. Energy intake at competition was similar for our subject and previous reports (1712 vs. 1739 kcals/day) [1]. However, prior investigations only monitored dietary intake for short periods of time, and many did not include information on dietary supplement use [1], thus limiting our ability to compare our more detailed analysis to previous reports.

Following the competition, our subject slowly increased energy intake. This was in an effort to limit a rapid increase in fat mass due to the known propensity for fat accumulation following energy restriction [28]. To our knowledge only one previous study assessed dietary intake following competition in female physique athletes. Walberg-Rankin et al. instructed female bodybuilders to keep 3-day food records the day of until 2 days following the competition and 19 to 21 days following the competition [14]. Compared with their participants’ energy intakes in the 1 month prior to the competition (1536–1839 kcals/day), energy intake immediately post competition (3237 kcals) and 3 weeks post was significantly greater (2790 kcals). This was associated with a rapid increase in body mass that was 1.2 kg above their initially reported body mass 1 month before the competition. Our subject was more cautious in the REC period than the athletes previously studied by diligently increasing energy intake slowly and limiting days ‘off’ the diet.

#### 4.1.2. Macronutrient Intake

During PREP, carbohydrate and fat intake decreased and protein intake increased compared to the subject’s baseline dietary habits. Carbohydrate intake fell below the Acceptable Macronutrient Distribution Range (AMDR) of 45%–65% of total caloric intake and protein intake rose above the AMDR of 10%–35%. Our subject’s macronutrient intake is consistent with previous reports in male and female physique competitors [1,11,14,20] which show carbohydrate intake below sports nutrition recommendations (e.g., 3–12 g/kg/day depending on training volume/intensity and body composition goals) [29] and protein intake above recommendations for strength training athletes (e.g., 1.2–2.0 g/kg/day) [29]. The elevated protein intake is presumably in an effort to maintain muscle mass (which our subject was successful at doing) during a period of weight loss, and is in line with recent findings demonstrating the efficacy of an increased protein intake during periods of energy restriction [30,31,32]. Following the competition, increases in energy intake were due to higher carbohydrate and fat intake. Protein intake decreased slightly, but still remained above 2.0 g/kg.

#### 4.1.3. Fiber Intake

Fiber intake decreased during PREP, but remained above the Dietary Reference Intake of 14 g/1000 kcals [33], and increased during REC. This was due to a reliance on nutrient-dense, low-energy foods such as fruits and vegetables [34]. High intake of fibrous foods likely promoted feelings of fullness and enabled this competitor to adhere to the energy restriction [35,36,37,38,39]. In addition, high fruit and vegetable intake is beneficial in ensuring that micronutrient needs are met while energy intake is reduced, and therefore should be included in the development of nutrition recommendations for competition PREP [34,36]. Prior studies in physique competitors have not quantified fiber consumption or tracked dietary intake as accurately so it is unknown if this is typical practice amongst physique competitors.

### 4.2. Exercise Training and Body Mass and Composition

Exercise training consisted of a high-volume resistance training regimen and modest amounts of aerobic exercise. Contrary to previous reports from the 1990s [14,16,40], but in agreement with more recent data [1,11] alterations in body mass and composition occurred mainly by reduced energy intake and not increased aerobic exercise. This may be indicative of an overall shift in preparation strategy over time. However, anecdotally we have observed that some current day physique athletes do rely on high levels of aerobic exercise to decrease fat mass before competitions. 

Unsurprisingly, 20-weeks of caloric restriction during PREP resulted in reduced body mass and fat mass, which was reversed when caloric intake increased during the 20-week REC phase. Lean mass was maintained during the PREP phase, likely due to a combination of high protein intake and the intensive resistance training program [30,31]. Our subject was lean (~15% body fat) at the start of competition preparation and achieved a body composition (~8%) similar to female bodybuilders previously studied [1,14,16]. While these reported body composition values are less than recommendations for essential fat for women [24], it is likely necessary in order to be competitive in this sport. As noted in the dietary intake section, the REC period has not been well studied in female physique competitors. Given the rapid increase in body mass (which overshot initial body mass) seen in the Walberg-Rankin et al. study [14], and an even more pronounced increase of 8.6 kg gain in body mass by 4 weeks post competition detected by Lamar-Hildebrand et al. in college-aged female bodybuilders [41] compared with the more controlled return to habitual body mass and composition in our subject, the REC period is deserving of additional investigation and likely requires unique dietary intake recommendations. 

### 4.3. Energy Availability and Menstrual Cycle

The EA of our subject fell below recommended levels of 30 kcal/kg of FFM [27] upon initiation of PREP and remained below this level for the entire 20 weeks. In addition, disruption to normal menstruation was reported early and amenorrhea occurred by the end of PREP. Due to an increase in energy intake and a decline in exercise energy expenditure during REC, EA increased to >35 kcal/kg FFM by week 10 of REC. Despite the return of caloric intake and body composition and mass to baseline levels during the 20-week REC period, menses did not resume until 71 weeks following the competition. Interestingly, the subject had returned to our laboratory for assessment of body composition (via DXA) 32 weeks post competition (i.e., 1 year since the initiation of PREP) and at week 71 post competition when menses resumed. Body composition and mass at those two assessment time points (20% body fat, 57.3 kg and 20% body fat, 56.1 kg, respectively) were higher than the habitual body composition and mass (~15% body fat, 54.9 kg) our subject had maintained for years leading up to the competition. 

This finding suggest that the reductions to EA and body composition which occur with preparation for physique competitions may have prolonged, detrimental effects on normal reproductive hormonal profiles. Resumption of menses may require that EA and body composition exceed baseline values for a prolonged period of time. Since our subject was concerned about the health implications of amenorrhea, she did not have plans to compete again. However, for competitors who plan to complete yearly, they may not have adequate time in between PREP cycles for menstrual cycle recovery. Prior investigations have noted that both resistance training and energy restriction are associated with alterations to reproductive hormones [14,42,43,44] leading to menstrual disruption (e.g., increases in estradiol and beta-endorphin which then reduce gonadotropin releasing hormone and luteinizing hormone pulsatility). Therefore, since physique athletes subject themselves to both intensive resistance training regimens and prolonged caloric restriction, they are at greater risk for menstrual disturbance and the associated insults to bone and metabolic health [21,22]. This has important ethical implications for the advice and treatment provided by coaches and health care professionals who work with these athletes.

Previous research on female athletes has established that those in lean build sports are more likely to have menstrual dysfunction than those in non-lean build sports [45]. However, while more common in this group of athletes than non-athletes, participation in physique competitions does not always lead to menstrual dysfunction [14,46]. Therefore, investigation of individual factors contributing to disruptions of normal menses, as well as analysis of alterations in reproductive hormones during PREP and REC warrant investigation in this at-risk group of athletes.

### 4.4. Strengths and Limitations

The current study has several strengths. Most notably this is the first to provide detailed, weighed dietary intake analysis over an extended time period (e.g., 40 weeks). This overcomes limitations of previous reports that rely solely on 1–3-day food records or food frequency questionnaires kept for a limited duration leading up to competitions [1]. Second, we accounted for intake of dietary supplementation, which has not been consistently reported in many earlier papers describing the dietary habits of physique competitors [1]. Third, we utilized DXA technology to evaluate changes in body composition. While we did rely on two separate DXA machines due to unavoidable equipment upgrades in our laboratory, the estimates tracked similarly with skinfold estimates, giving us greater confidence in our measurements. Fourth, this study is the first to estimate EA and track menses during PREP and REC. These are important considerations for the development of sports nutrition recommendations that will also support the long-term health of physique athletes. Finally, the case study approach is also a strength since longer-term, detailed information was obtained which would be challenging with a larger cohort [47]. These findings can be utilized to inform future studies in this athletic population.

Despite these strengths, we acknowledge limitations of this study. First, we did not collect biochemical or clinical data (aside from body composition). Future investigations should be done prospectively and plan to obtain blood and urine samples to evaluate alterations in hormonal and metabolite values related to weight loss/gain and menstrual function as well as relevant clinical outcomes (e.g., metabolic rate, blood pressure, heart rate, etc.). Second, we did not include psychological measures. Questionnaires related to dietary restraint, disinhibition, and disordered eating would be valuable to include in future longitudinal research on physique competitors. Third, our participant did not track timing of dietary intake and supplement use throughout the day, or in relation to workouts. While nutrient timing is an important and interesting sports nutrition consideration, this level of detail would likely be unrealistic in investigations of similar duration. Nonetheless, future trials and interventions focusing on this topic may provide important information on nutrient timing strategies to assist physique athletes in achieving their body composition and/or strength goals before and after competitions. 

### 4.5. Future Directions

The popularity of physique competitions is increasing, with a greater number of organizations created and competitions held each year [48]. Therefore, research is needed in order to establish evidence-based nutrition and exercise recommendations related to improving performance, while minimizing potential adverse health outcomes of caloric restriction in these athletes. Randomized-controlled trials will likely be unfeasible in this population. Instead, long-term observational trials which track competitors during PREP and REC phases will be needed. Specific questions to answer include: What is the typical degree of caloric restriction competitors subject themselves to? What is the prevalence of the female athlete triad? What best predicts both performance and maintenance of health in these athletes (e.g., baseline caloric intake, degree of restriction employed, age, dietary composition, exercise energy expenditure, etc.)? What is the time course for recovery of physiological, metabolic, and menstrual responses to competition preparation? What is the long-term impact of several cycles of competition preparation and recovery on health outcomes? What are the psychological ramifications (e.g., eating attitudes, mood disturbance, and sleep habits) of physique competition participation? Overall, these competitors are a unique and understudied group of athletes whom much can be learned from in regards to the metabolic adaptations to caloric restriction during competition preparation and metabolic recovery during refeeding following competitions.

## 5. Conclusions

This case study provides the first long-term assessment of dietary intake, body mass/composition, and menstrual cycle changes associated with competition PREP and REC in a drug-free, female figure competitor. As expected caloric restriction and decreased EA led to a decline in fat and body mass and cessation of menses. Energy intake, body mass and fat mass returned to baseline levels by the end of the 20-week REC period. However, return of menstruation was delayed, not resuming until over a year following the competition. Our case study adds long-term, detailed information to the limited literature available on this population. Future studies should build upon this approach in order to lead to the creation of evidence-based dietary intake and exercise recommendations for physique competitors across the competitive cycle that aims to increase ‘performance’ (e.g., subjectively rated appearance) while maintaining the health of the competitors.

## Figures and Tables

**Table 1:** Sample Weighed Food Record Representative of a Typical Day During both PREP and REC.

Food ^1^	Preparation and/or Description	Weight (g)
		(Approximate Volume)
Breakfast:		
Oatmeal	With water	40 (dry)
		(1/2 cup, dry)
Whey Protein Isolate (De Novo Nutrition)	N/A	20
		(2/3 scoop)
Blueberries	Frozen, no sugar added	70
		(1/3 cup)
Peanut Butter	Natural, no added oil, sugar, or salt	15
		(1 Tbsp)
Morning Snacks:		
Greek Yogurt (Chobani)	Plain, non-fat	150
		(1 single-serve container)
Apple	Raw, with peel	180
		(1 medium, 3-inch diameter)
Almonds	Raw, unsalted	12
		(12 almonds)
Lunch:		
Broccoli	Steamed from fresh or frozen	142
		(1 small stalk)
Black Beans	Canned, drained and rinsed	120
		(1/2 cup)
Brown Rice, Jasmine	With water, no added oil or salt	98 (prepared)
		(1/2 cup, prepared)
Hummus (Sabra)	Classic flavor	28
		(2 Tbsp)
Whey Protein Isolate (De Novo Nutrition)	N/A	20
		(2/3 scoop)
Afternoon Snacks:		
Greek Yogurt (Chobani)	Plain, non-fat	150
		(1 single-serve container)
Blueberries	Frozen, no sugar added	70
		(1/3 cup)
Oatmeal	With water	40 (dry)
		(1/2 cup, dry)
Whey Protein Isolate (De Novo Nutrition)	N/A	20
		(2/3 scoop)
Green Bell Pepper	Raw	164
		(1 large, 3-inch diameter)
Dinner:		
Tilapia fillet	Baked, from frozen	114
		(1.3 fillets)
Green Bell Pepper	Raw	164
		(1 large, 3-inch diameter)
Kale	Raw	100
		(6 cups, loosely packed)
Carrot	Raw	100
		(0.9 cups, grated)
Red Cabbage	Raw	100
		(1.1 cups, chopped)
Extra Virgin Olive Oil	Dressing for kale salad	10
		(2 tsp)
Sesame Seed Oil	Dressing for kale salad	5
		(1 tsp)
Rice Vinegar	Dressing for kale salad	15
		(1 Tbsp)
Sesame Seeds	Whole, dry, dressing for kale salad	5
		(1/2 Tbsp)
Brown Rice, Jasmine	With water, no added oil or salt	80 (prepared)
		(2/5 cup, prepared)
Evening Snacks:		
Almond Butter	Natural, no added oils, sugar, or salt	18
		(1 Tbsp)
Beverage Intake ^2^:		
Water	N/A	24–48 fl. oz.
Diet Soda	N/A	24–36 fl. oz.
Coffee/tea	Black, unsweetened	24–48 fl. oz.

PREP: Competition Preparation; REC: Competition recovery; Tbsp: Tablespoon. ^1^ Use of seasonings (e.g., salt) was not weighed or tracked; ^2^ Beverage intake was not weighed or rigidly tracked. It was reported by participant as typical consumption.

**Table 2:** Energy and Macronutrient Intake.

	Energy (kcals/Day)	CHO (g)	Protein (g)	Fat (g)	Fiber (g)
		(% Total kcals)	(% Total kcals)	(% Total kcals)	
		(g/kg BM)	(g/kg BM)		
Baseline	2010	225	120	70	48
		45%	24%	31%	
		(4.1 g/kg)	(2.2 g/kg)		
Week 1 PREP	1798	187	150	50	40
		42%	33%	25%	
		(3.4 g/kg)	(2.7 g/kg)		
Week 10 PREP	1541	143	150	41	24
		37%	39%	24%	
		(2.7 g/kg)	(2.9 g/kg)		
Week 20 PREP	1712	188	150	40	34
		44%	35%	21%	
		(3.8 g/kg)	(3.0g/kg)		
Week 10 REC	2032	219	146	63	49
		43%	29%	28%	
		(4.2 g/kg)	(2.8 g/kg)		
Week 20 REC	2023	233	133	62	47
		46%	26%	28%	
		(4.2g/kg)	(2.4 g/kg)		

PREP: Competition preparation; REC: Competition recovery; CHO: carbohydrate; BM: body mass.
